# Exploring the assessment of post-cardiac valve surgery pulmonary complication risks through the integration of wearable continuous physiological and clinical data

**DOI:** 10.1186/s12911-025-02875-2

**Published:** 2025-01-31

**Authors:** Lixuan Li, Yuekong Hu, Zhicheng Yang, Zeruxin Luo, Jiachen Wang, Wenqing Wang, Xiaoli Liu, Yuqiang Wang, Yong Fan, Pengming Yu, Zhengbo Zhang

**Affiliations:** 1 Medical Innovation Research Division, Chinese PLA General Hospital, Beijing, China; 2 Department of Rehabilitation Medicine, West China Tianfu Hospital, Sichuan University, Chengdu, China; 3 PAII Inc, Palo Alto, CA USA; 4 Department of Rehabilitation Medicine Center, West China Hospital, Sichuan University, Chengdu, China; 5 General Hospital of Tibet Military Region, Lhasa, China; 6 Department of Cardiovascular Surgery, West China Hospital, Sichuan University, Chengdu, China

**Keywords:** Postoperative pulmonary complications, Wearable devices, Continuous physiological signals, Heart valve surgery, Preoperative assessment

## Abstract

**Background:**

Postoperative pulmonary complications (PPCs) following cardiac valvular surgery are characterized by high morbidity, mortality, and economic cost. This study leverages wearable technology and machine learning algorithms to preoperatively identify high-risk individuals, thereby enhancing clinical decision-making for the mitigation of PPCs.

**Methods:**

A prospective study was conducted at the Department of Cardiovascular Surgery of West China Hospital, Sichuan University, from August 2021 to December 2022. We examined 100 cardiac valvular surgery patients, where wearable technology was utilized to collect and analyze nocturnal physiological data at the 24-hour admission, in conjunction with clinical data extraction from the Hospital Information System’s electronic records. We systematically evaluated three different input types (physiological, clinical, and both) and five classifiers (XGB, LR, RF, SVM, KNN) to identify the combination with strong predictive performance for PPCs. Feature selection was conducted using Recursive Feature Elimination with Cross-Validated (RFECV) for each model, yielding an optimal feature subset for each, followed by a grid search to tune hyperparameters. Stratified 5-fold cross-validation was used to evaluate the generalization performance. The significance of AUC differences between models was tested using the DeLong test to determine the optimal prognostic model comprehensively. Additionally, univariate logistic regression analysis was conducted on the features of the best-performing model to understand the impact of individual feature on PPCs.

**Results:**

In this study, 22 patients (22%) developed PPCs. Across classifiers, models combining both physiological and clinical features performed better than physiological or clinical features alone. Specifically, including physiological data in the classification model improved AUC, ACC, F1, and precision by an average of 8.32%, 1.80%, 3.28% and 6.06% compared to using clinical data only. The XGB classifier, utilizing both dataset, achieved the highest performance with an AUC of 0.82 (± 0.08) and identified eight significant features. The DeLong test indicated that the XGB model utilizing the both dataset significantly outperformed the XGB models trained on the physiological or clinical datasets alone. Univariate logistic regression analysis suggested that surgical methods, age, nni_50, and min_ven_in_mean are significantly associated with the occurrence of PPCs.

**Conclusion:**

The integration of continuous wearable physiological and clinical data significantly improves preoperative risk assessment for PPCs, which helps to optimize surgical management and reduce PPCs morbidity and mortality.

**Supplementary Information:**

The online version contains supplementary material available at 10.1186/s12911-025-02875-2.

## Introduction

Postoperative pulmonary complications (PPCs) are a significant concern following heart surgeries, associated with increased morbidity, prolonged hospital stay, and mortality [1]. With more than 40 million people afflicted with mitral or aortic valve disease and over 180,000 heart valve replacement surgeries performed annually, the prevalence of PPCs remains a critical issue in cardiac surgery patients [2]. While cardiac valve surgery patients face a greater risk due to specific surgical sites and procedures, PPCs incidence can still surge to 23% in non-cardiothoracic surgeries [3]. Preoperative pulmonary evaluation presents a promising approach to identify patients at risk for PPCs, guide decision-making for interventions, and minimize the adverse effects of surgery [4].

Despite the availability of high-scoring models for identifying individuals at high risk for PPCs, current methods face limitations in guiding preoperative interventions. Several risk scoring tools focus on a single pulmonary complication, such as “respiratory failure risk index” for respiratory failure [5], “postoperative pneumonia risk index” for pneumonia [6], “surgical lung injury prediction” for postoperative acute lung injury [7], which do not offer a comprehensive analysis of lung risks in the complex pulmonary complications situation. While studies like ARISCAT [8] and LasVegas [9] employ comprehensive outcome measures for PPCs, their inclusion of intraoperative factors restricts their predictive utility to a preoperative setting. The advent of artificial intelligence algorithms has led to machine learning models that demonstrate impressive predictive capabilities in prediction of PPCs risk [10, 11]. Unfortunately, these studies have also utilized intraoperative factors, causing an inability to assess patient risk before surgery. Notably, Bing Xue established a prediction model using preoperative electronic medical record information alone through machine learning algorithms, whose performance is much lower than the model using both preoperative and intraoperative information [10]. Predicting postoperative pulmonary complications before surgery requires more patient information, and using preoperative clinical information alone is insufficient.

Compared to the discrete data of electronic medical records, the paradigm of wearable technology integrated with artificial intelligence(AI) potentially offers new insights for addressing this issue. Wearable devices provide a continuous and multidimensional monitoring of vital signs in both inpatient and remote settings, enabling a noninvasive patient assessment with minimal user input. Amidst the epidemic’s mandated push, notable advancements have been achieved in wearable device clinical applications, including utilizing sensors to predict individualized lab measurements [12], and exploring wearables’ role in remote cardiovascular screening, diagnosis, and management [13]. These advancements underscore the value of wearable devices for continuous and longitudinal clinical assessment. We hypothesize that wearable devices, with their ability to monitor various physiological parameters continuously, could significantly enhance the preoperative risk assessment for PPCs. Factors such as respiratory function, type of surgery, and diaphragmatic dysfunction have been identified to associate with PPCs [14]. Sleep disturbances and deprivation, particularly, emerges as significant factors, with ties to increased anesthesia and postoperative complications. For instance, obstructive sleep apnea is recognized as a preoperative PPCs risk factor [15]. Wearable devices effectively monitor sleep, demonstrating high concordance with polysomnography [16] and improved precision in extreme vital signs, with less measurement bias [17]. To our knowledge, this study first assesses the PPCs risk of heart valve patients based on wearable continuous physiological during nocturnal sleep and clinical data before surgery. It is expected to assist doctors in identifying high-risk populations before surgery and provide guidance for preoperative interventions.

## Methods

### Design and participants

A prospective study was conducted at the Department of Cardiac and Major Vascular Surgery of West China Hospital, Sichuan University in adherence to the principles outlined in the Declaration of Helsinki [18], from August 2021 to September 2022. This study was approved by the Ethics Committee of the West China Hospital of Sichuan University, Ethics No.20,211,023, Clinical Registration No. ChiCTR2100050005 (http://www.chictr.org.cn). All participants provided written informed consent, affirming their full comprehension and voluntary participation in the study. All personal information collected during the study is kept confidential and not disclosed to third parties without participants’ consent.

Inclusion criteria: (1) patients undergoing elective cardiac valve surgery; (2) aged 18 or above; (3) no rehabilitation training or other intervention before surgery; (4) agreeing to participate in the study and sign informed consent; (5) wearing data collection equipment throughout the process.

Exclusion criteria: (1) emergency surgery; (2) refusal to participate in the trial; (3) consciousness dysfunction; (4) expected life less than six months; (5) participation in other drug or device clinical trials have not reached the endpoint before inclusion; (6) poor compliance and failure to complete the study as required.

### Data collection

Continuous physiological data, including electrocardiograph(ECG), SpO_2_, respiratory signals from the chest and abdomen, and triaxial acceleration signals, were collected from patients with heart valve disease at the 24-hour admission using a medical-grade portable monitoring device (SensEcho ^®^). SensEcho consists of a flexible vest and a signal acquisition terminal embedded in it. The fabric electrodes embedded in the vest enable the collection of single-lead ECG signals. The sensor coils located on the chest and abdomen acquire chest/abdomen respiratory signals through respiratory inductive plethysmography. The accelerometer sensor integrated in the terminal collected posture/body movement signals. A ring worn on the thumb collected blood oxygen signals in real-time and transmits them to the terminal via Bluetooth synchronization [19]. The physiological signal collection process lasted for 24 h, during which a senior physiotherapist operated SensEcho. After the completion, the data files were exported for subsequent analysis. This device has been validated in our previous clinical studies for monitoring sleep apnea [20], and six-minute walk test [21].

Medical electronic case information was collected from the Cardiac Surgery Database by the investigators using a data collection form, including demographics, diagnosis, preoperative history, examinations, and surgical methods.

### Outcome

PPCs, were diagnosed in 14 days postoperatively according to the Melbourne Group Scale (MGS) [22] listed in Table 1.

**Table 1:** Melbourne Group Scale Evaluation Criteria

At least the following four items can be determined to occur PPCs:	
1.	Chest radiograph report of collapse/consolidation;
2.	Leukocyte cell count > 11.2 × 10^9^/L or prescription of an antibiotic specific for respiratory infection (except for those routinely used after surgery);
3.	Oral temperature > 38 °C, without fever caused by reasons other than lung;
4.	Microbiological evidence of sputum (+);
5.	Yellow or green sputum different from preoperative assessment;
6.	SpO_2_ is < 90% in indoor environment;
7.	Clinical diagnosis of pneumonia or pulmonary infection;
8.	Stay in the care unit for > 36 h or enter the care unit again due to respiratory problems.

*PPCs*, Postoperative pulmonary complications

### Data pre-processing

We extracted continuous physiological data from the nighttime sleep phase to characterize the individual’s essential state. The raw physiological signals were smoothed using a moving average filter. Outliers exceeding three standard deviations are identified and removed from the signal. Hamilton’s method [23]was used to detect R peaks of ECG signals. Khodadad’s method [24] was used to detect peaks and valley values of respiratory signals. Moreover, the physiological data quality for each patient was assessed by the ECG and respiratory signal quality assessment algorithms [25] combined with expert experience. Data will be excluded with the following situations: sleep duration less than 4 h, poor signal quality (available ECG less than 50%, available blood oxygen less than 50%, etc.), equipment failure (frequent packet loss, signal loss, etc.), severe baseline drift, high noise impact, etc.

### Feature extraction

**Fig. 1:**
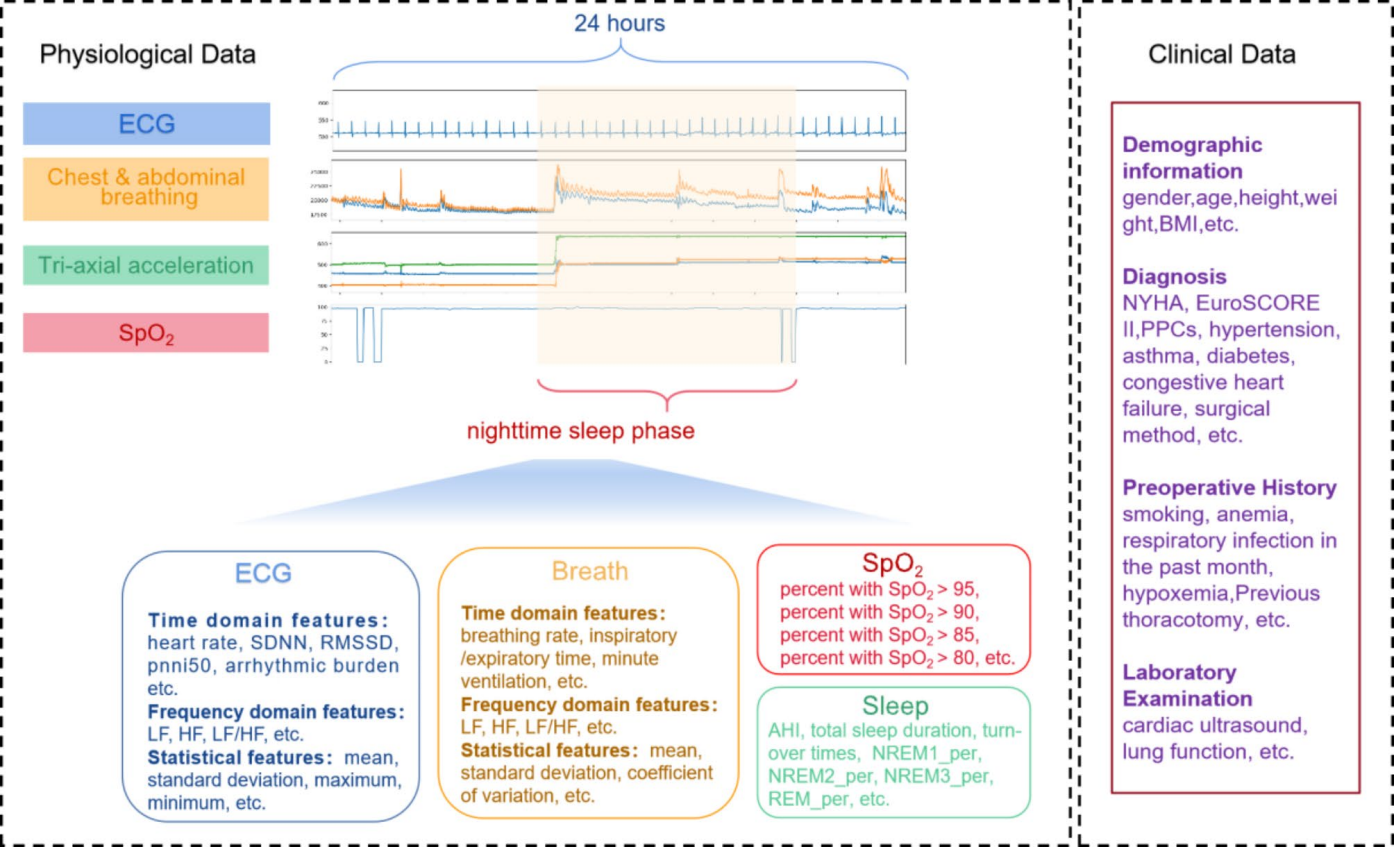
Schematic of feature extraction. The fragment lengths are not the full 24-hour or nocturnal sleep stages, and the screenshots of physiological signals in the figure are illustrative only. *SDNN*, the standard deviation of the normal heart beats (RR intervals); *RMSSD*, the square root of the mean of the sum of successive differences between adjacent RR intervals; *pnni50*, the proportion of RR intervals greater than 50ms, out of the total number of RR intervals; *LF*, low frequency; *HF*, high frequency; arrhythmic burden, the proportion of the number of the difference between the two RR intervals greater than 145 ms in the total number of RR intervals; *NREM1_per*, percentage of NREM (Non-Rapid Eye Movement)1 sleep periods; *NREM2_per*, percentage of NREM2 sleep periods; *NREM3_per*, percentage of NREM3 sleep periods; *REM_per*, percentage of REM (Rapid Eye Movement) sleep periods; *AHI*, apnea-hypopnea index; *BMI*, body mass index; *NYHA*, New York Heart Association; *EuroSCORE II*, the European System for Cardiac Operative Risk Evaluation II; *PPCs*, postoperative pulmonary complications; *TAVR*, transcatheter aortic valve replacement; *SAVR*, surgical aortic valve replacement

In this paper, we extracted 45 physiological features from continuous physiological signals covering relevant aspects such as ECG, respiration, blood oxygenation and sleep, and 45 clinical features from electronic medical records, including demographic information, clinician diagnosis, preoperative history, examinations and surgical methods. The accuracy and reliability of sleep feature calculation methods have been validated [20]. Extract the temporal and frequency-domain features of the physiological signals. Additionally, calculate statistical metrics such as the mean, standard deviation, maximum, minimum, coefficient of variation for the temporal features to gain further insights. The feature extraction process is shown in Fig. 1.

### Model

**Fig. 2:**
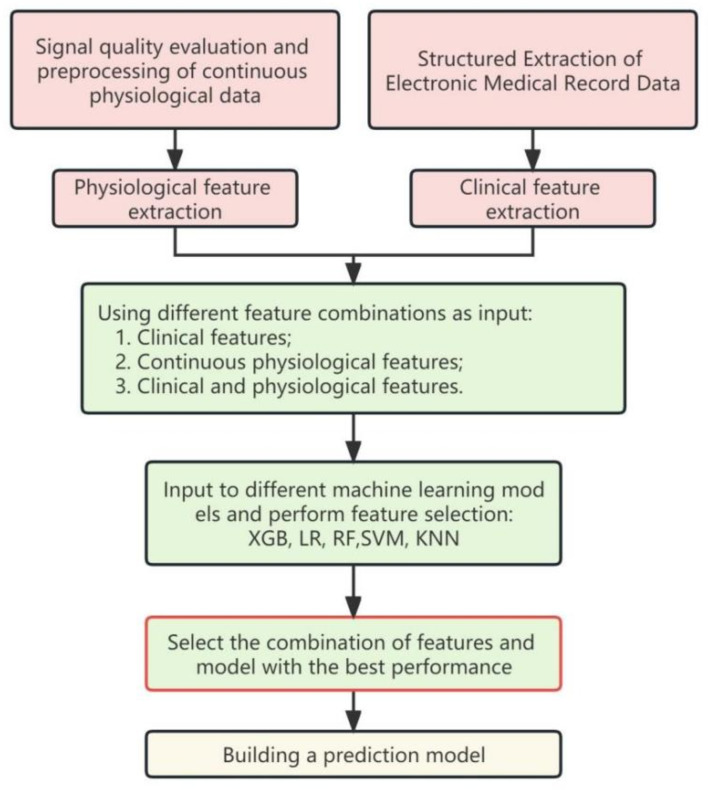
Modelling procedure. *XGB*, XGBoost; *LR*, Logistic Regression; *RF*, Random Forests; *SVM*, Support Vector Machine; *KNN*, k-Nearest Neighbor

After data pre-processing and feature extraction, physiological and clinical features were obtained. Try different feature combinations as input separately: physiological, clinical, and both (physiological combined with clinical) datasets. Each input combination are experimented with various machine learning classifiers, including XGBoost (XGB), Logistic Regression (LR), Random Forest (RF), Support Vector Machine (SVM), and k-Nearest Neighbor (KNN), to build powerful PPCs risk prediction models. The model with the best performance was ultimately chosen for identifying PPC risks. See Fig. 2 for details. Stratified 5-fold cross-validation was used to evaluate the generalization performance considering the imbalance between positive and negative samples. The predictive ability was evaluated from multiple perspectives by calculating the area under the ROC curve (AUC), accuracy (ACC), F1 score (F1) and precision.

Furthermore, the Delong test was used to measure the differences between models based on both datasets and those based on physiological or clinical datasets only. Univariate logistic regression was conducted on the optimal feature subset of the best-performing model to examine the impact of individual features on the outcome. All statistical analyses were conducted using Python Version 3.7 with the libraries scikit-learn 0.22.1 and XGBoost 1.2.0. A Windows 10 desktop with an Intel Core i7 processor (2.8 GHz, 8 cores) was used for data analysis.

## Results

### Postoperative pulmonary complications

**Fig. 3:**
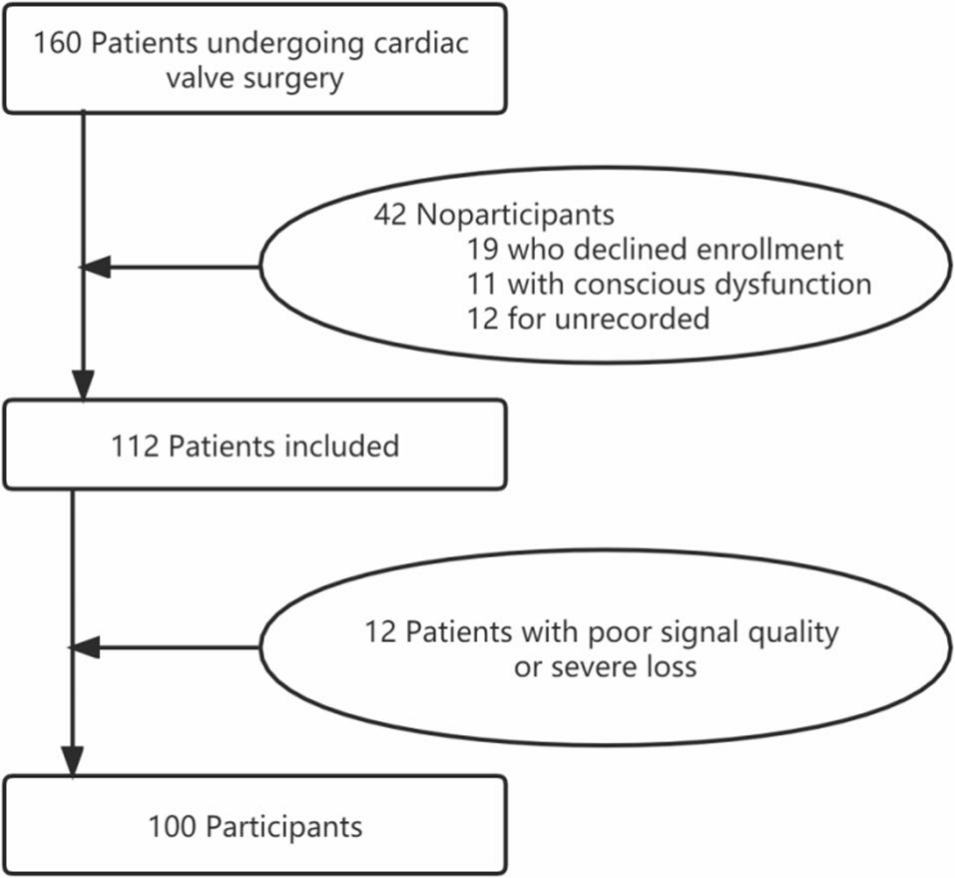
Recruitment flowchart

Among 112 eligible patients, 100 had physiological signals that met the quality requirements (Fig. 3), which are involved in the analysis. No unexpected events or study withdrawals were reported throughout the study. Among patients, 68% were male, with a mean age of 64.35, a mean BMI of 23.62, a mean NYHA classification of 2.52, and a mean EuroSCORE II of 4.97. 54% of the patients underwent TAVR surgery, while the remaining patients underwent SVAR surgery. According to the Melbourne Group Scale, 22 patients (22%) developed PPCs. The demographic and surgical characteristics are detailed in Table 2.

**Table 2:** Demographic data

Variable	PPCs (*N* = 22)	Non-PPCs (*N* = 78)	Total (*N* = 100)
Demographics			
Gender (male), n (%)	13 (59.0)	55 (70.5)	68 (68)
Age (years), mean ± SD	57.77 ± 10.54	66.21 ± 11.62	64.35 ± 11.87
Height (cm), mean ± SD	160.95 ± 8.45	160.59 ± 8.15	145.67 ± 8.18
Weight (kg), mean ± SD	62.75 ± 15.53	60.59 ± 10.08	61.07 ± 11.45
BMI, mean ± SD	24.11 ± 4.97	23.48 ± 3.35	23.62 ± 3.75
Principal Diagnosis, n (%)			
NYHA classification			
II	11 (50.0)	40 (51.3)	51 (51)
III	10 (45.5)	36 (46.2)	46 (46)
IV	1 (4.5)	2 (2.5)	3 (3)
EuroSCORE II, mean ± SD	4.36 ± 3.32	5.14 ± 2.80	4.97 ± 2.92
Hypertension	8 (36.3)	39 (50)	47 (47)
Preoperative smoking history	6 (27.2)	20 (25.6)	26 (26)
Preoperative anemia	1 (4.5)	3 (3.8)	4 (4)
Respiratory infection in the past month	1(4.5)	2 (2.5)	3 (3)
Congestive heart failure	16 (72.7)	64 (82.1)	80 (80)
Preoperative hypoxemia	6 (27.2)	28 (35.9)	34 (34)
Previous thoracotomy	2 (9.0)	5 (6.4)	7 (7)
Surgical method, n (%)			
TAVR	3 (13.6)	51 (65.3)	54 (54)
SAVR	19 (86.3)	27 (34.6)	46 (46)

Notes: Demographic data is given as ‘mean ± standard deviation’ or ‘N (%)’, as appropriate. *PPCs*, postoperative pulmonary complications; *SD*, standard deviation; *BMI*, body mass index; *NYHA*, New York Heart Association; *EuroSCORE II*, the European System for Cardiac Operative Risk Evaluation II; *TAVR*, transcatheter aortic valve replacement; *SAVR*, surgical aortic valve replacement

### Model performance

Feature selection was conducted on 45 physiological and 45 clinical features, resulting in distinct optimal feature subsets for each classifier. Each model was then fine-tuned for its specific optimal subset of features. We explored the predictive performance of different combinations (physiological, clinical, and both) and models (XGB, LR, RF, SVM, KNN), as detailed in Table 3. The P-value represented the results of the DeLong test, comparing the model based on the both dataset against those using the clinical or physiological dataset individually.

**Table 3:** Comparison of model performance for different feature combinations

Model	Feature combination	AUC	ACC	F 1	Precision	*P*-Value^a^
**XGB**	Physiological	0.69 ± 0.15	0.79 ± 0.06	0.73 ± 0.07	0.74 ± 0.14	0.01*
	Clinical	0.78 ± 0.08	0.77 ± 0.03	0.68 ± 0.03	0.61 ± 0.04	0.05*
	**Both**	**0.82 ± 0.08**	**0.80 ± 0.01**	**0.74 ± 0.0**	**0.75 ± 0.10**	**/**
LR	Physiological	0.60 ± 0.08	0.75 ± 0.05	0.67 ± 0.02	0.60 ± 0.02	0.17
	Clinical	0.75 ± 0.11	**0.78 ± 0.03**	**0.70 ± 0.04**	**0.66 ± 0.11**	0.65
	Both	**0.77 ± 0.09**	0.75 ± 0.05	0.69 ± 0.06	0.65 ± 0.09	**/**
RF	Physiological	0.63 ± 0.07	0.76 ± 0.04	0.67 ± 0.04	0.61 ± 0.04	0.00*
	Clinical	0.73 ± 0.03	0.77 ± 0.05	0.71 ± 0.03	0.69 ± 0.09	0.08
	Both	**0.80 ± 0.10**	**0.80 ± 0.04**	**0.75 ± 0.04**	**0.77 ± 0.10**	**/**
SVM	Physiological	0.64 ± 0.15	0.77 ± 0.05	0.68 ± 0.05	0.61 ± 0.05	0.82
	Clinical	0.54 ± 0.29	0.76 ± 0.04	0.67 ± 0.04	0.61 ± 0.04	0.45
	Both	**0.77 ± 0.17**	**0.78 ± 0.03**	**0.68 ± 0.04**	**0.61 ± 0.04**	**/**
KNN	Physiological	0.42 ± 0.08	0.72 ± 0.06	0.65 ± 0.04	0.60 ± 0.04	0.00*
	Clinical	0.66 ± 0.13	0.71 ± 0.02	0.68 ± 0.02	0.65 ± 0.03	0.96
	Both	**0.70 ± 0.10**	**0.75 ± 0.06**	**0.73 ± 0.06**	**0.74 ± 0.08**	**/**

Notes: AUC, ACC, F1, and precision values were expressed as mean ± standard deviation of five-fold cross-validation. *AUC*, the area under the ROC curve; *ACC*, accuracy; *F1*, F1 score; XGB, XGBoost; LR, Logistic Regression; RF, Random Forests; SVM, Support Vector Machine; KNN, k-Nearest Neighbor

a. The p-value indicates the significance of performance differences between the model based on the both dataset against those using the clinical or physiological dataset individually, as evaluated by the DeLong test

* *p* < 0.05

Across different classifiers, models combining both physiological and clinical features tended yield better performance compared to physiological or clinical features alone. Including physiological data in the classification model improved AUC, ACC, F1, and precision by an average of 8.32%, 1.80%, 3.28%, 6.06% compared to using only clinical data. The DeLong test results showed that the XGB model utilizing the both dataset significantly outperformed the XGB models trained on the physiological or clinical datasets alone. Additionally, the Random Forest and K-Nearest Neighbors models that incorporated both datasets demonstrated a significant improvement in performance compared to those that relied solely on the physiological dataset. The results indicated that the integration of physiological and clinical data typically enhances the capability in preoperative evaluation of PPCs risk.

**Fig. 4:**
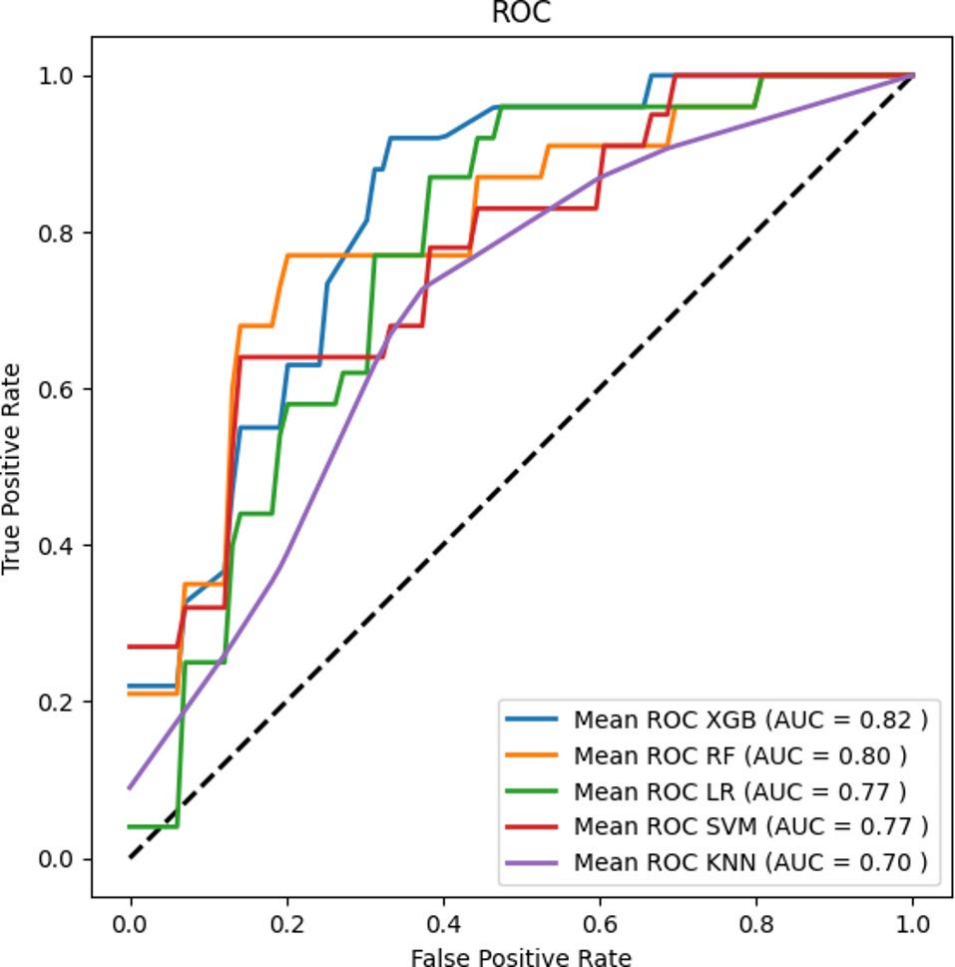
ROC curves for different machine learning models using both physiological and clinical features as inputs. The ROC curves for machine learning models were the average score of the results of a five-fold cross-validation. *XGB*, XGBoost; *LR*, Logistic Regression; *RF*, Random Forests; *SVM*, Support Vector Machine; *KNN*, k-Nearest Neighbor

When using physiological and clinical features as inputs, the XGB model achieved the highest AUC (0.82) and ACC (0.80), as well as relatively high F1 score (0.74) and precision (0.75) among models. This indicated that the XGB model demonstrates high overall performance and stability on the classification task, able to correctly identify most high PPCs risk events while maintaining a low false positive rate. The optimal hyperparameters for the XGB model were determined as follows: learning_rate = 0.1, n_estimators = 32, max_depth = 3, random_state = 1. The RF model had acceptable AUC and ACC, as well as the highest F1 score and precision. The model with the lowest AUC was KNN. Figure 4 compares different classifiers’ ROC curves using both physiological and clinical features as inputs.

**Fig. 5:**
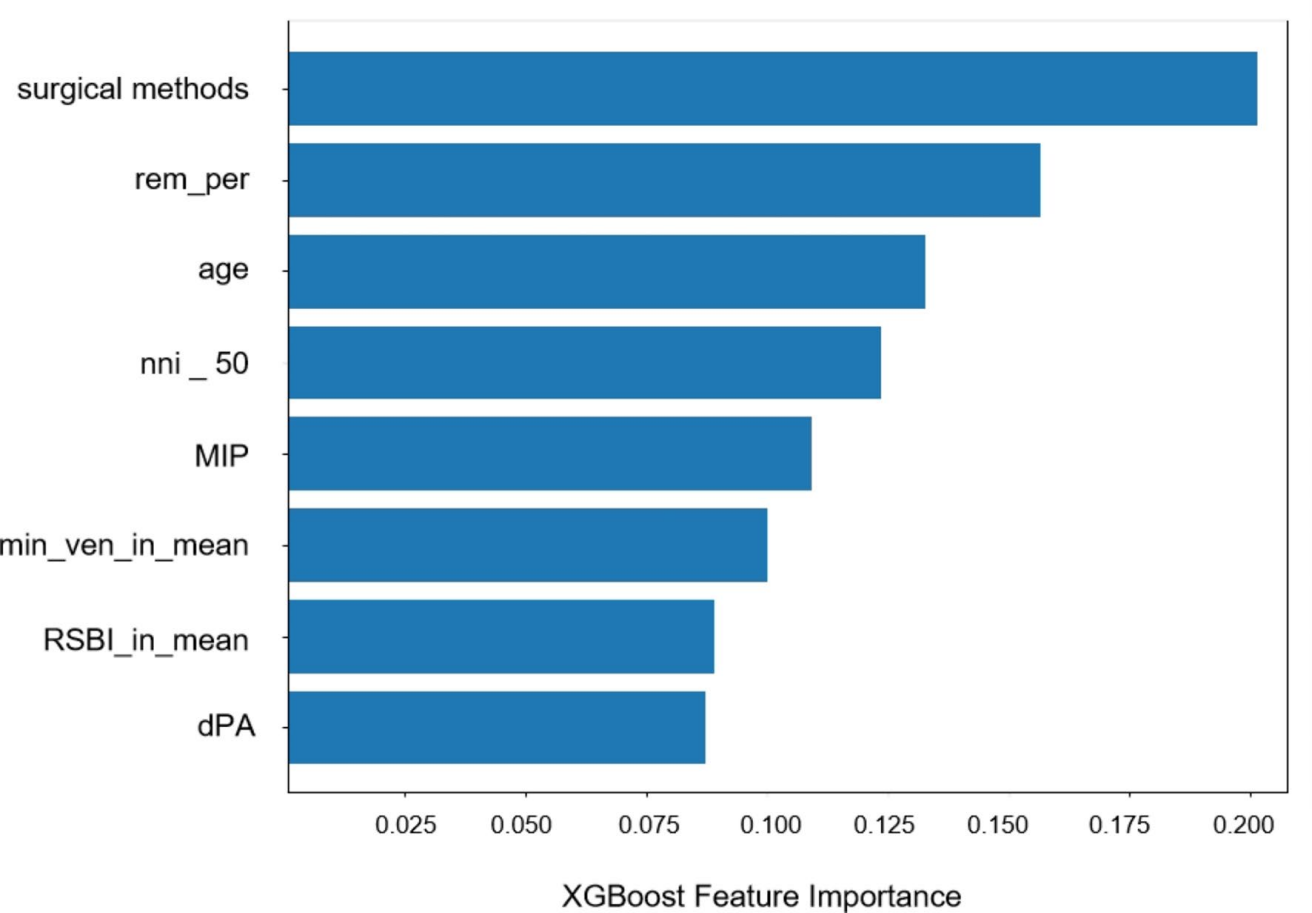
Importance ranking of the ten filtered features. *Surgical methods*, transcatheter aortic valve replacement (TAVR) and surgical aortic valve replacement (SAVR); *rem_per*, percentage of rapid eye movement (REM) sleep duration in total sleep; *age*, age of patient; *nni_50*, number of interval differences of successive normal heart beats greater than 50 ms; *MIP*, maximum inspiratory pressure; *min_ven_in_mean*, mean inspiratory minute ventilation; *RSBI_in_mean*, mean inspiratory rapid shallow breathing index (RSBI_in), RSBI_in is breath rate divided by inspiratory tidal volume; *dPA*, pulmonary artery diameter

The XGB model selected eight features, including four physiological features: RSBI_in_mean (the ratio of respiratory frequency to inspiratory tidal volume), min_ven_in_mean (mean inspiratory minute ventilation), rem_per (percentage of REM sleep duration in total sleep), nni _ 50 (number of intervals greater than 50ms between two NN), and four clinical features: surgical methods, age, MIP (maximum inspiratory pressure), dPA (pulmonary artery diameter, mm). Among features, the importance of surgical methods ranked first, indicating that different surgical methods have a significant impact on PPCs risk. In addition, the proportion of deep sleep was also an important risk factor for PPCs. Figure 5 shows the importance ranking of the ten features.

To further understand the individual contribution of each feature to PPC, we performed univariate logistic regression on the subset of 8 optimal features identified by the XGB model. The results, as presented in Table 4, revealed that all the features demonstrated AUCs exceeding 0.6, which underscored the predictive strength of this selected feature subset for PPCs. Notably, our analysis indicated that surgical methods, age, nni_50, and min_ven_in_mean were significantly associated with the occurrence of PPCs. This suggested that these features have a substantial influence on the ability to predict outcomes.

**Table 4:** Univariate logistic regression analysis of each feature in the optimal subset of the XGB model versus PPCs

Feature	AUC	95% CI	Sensitivity	Specificity	*p*-value
surgical methods	0.76	(0.63, 0.88)	0.86	0.65	0.00*
age	0.73	(0.60, 0.86)	0.68	0.77	0.01*
nni_50	0.67	(0.54, 0.81)	0.82	0.60	0.05*
min_ven_in_mean	0.67	(0.53, 0.80)	0.82	0.54	0.03*
rem_per	0.64	(0.50, 0.78)	0.46	0.89	0.12
dPA	0.64	(0.50, 0.77)	0.77	0.49	0.28
RSBI_in_mean	0.63	(0.49, 0.77)	0.64	0.69	0.16
MIP	0.60	(0.46, 0.74)	0.41	0.89	0.10

*XGB*, XGBoost; *PPCs*, Postoperative pulmonary complications; *Surgical methods*, transcatheter aortic valve replacement (TAVR) and surgical aortic valve replacement (SAVR); *rem_per*, percentage of rapid eye movement (REM) sleep duration in total sleep; *age*, age of patient; *nni_50*, number of interval differences of successive normal heart beats greater than 50 ms; *MIP*, maximum inspiratory pressure; *min_ven_in_mean*, mean inspiratory minute ventilation; *RSBI_in_mean*, mean inspiratory rapid shallow breathing index (RSBI_in), RSBI_in is breath rate divided by inspiratory tidal volume; *dPA*, pulmonary artery diameter. * *p* < 0.05

## Discussion

Although there have been a series of studies on risk prediction models of PPCs, the complex factors of the disease and human body make the risk stratification of PPCs without a “one size fits all” model [3]. This is the first study to evaluate the risk of postoperative pulmonary complications in patients with heart valve disease using wearable continuous physiological data combined with clinical information before surgery. The results are encouraging, as the addition of continuous physiological state information captured by wearable devices improved the prediction of an individual’s risk of future pulmonary complications, demonstrating the potential value of wearable data in longitudinal prediction. Later sections discuss the incidence of PPCs in patients undergoing heart valve surgery, data inputs for preoperative assessment of PPCs risk, and a preoperative PPCs risk prediction model.

### High PPCs rate in patients undergoing cardiac valve surgery

PPCs have a high incidence rate and are more closely associated with postoperative mortality than cardiac complications [26]. In particular, in the realm of cardiac surgery with cardiopulmonary bypass (CPB), the incidence of PPCs was reported to be 6.96%, with pneumonia, respiratory failure, and reintubation at 5.45%, 3.11%, and 0.54%, respectively [27].

In this study, the pulmonary complication rate after heart valve surgery was 22%, higher than in other studies. The possible reasons are two-fold. One reason is that West China Hospital of Sichuan University admits patients with heart valve diseases who often have long disease durations, severe symptoms, complex conditions, and greater surgical difficulties. These factors may contribute to a relatively higher incidence rate of postoperative complications. Another reason is that different studies may have variations in the definition of PPCs and types of surgeries, leading to different rates of PPCs occurrence.

### Continuous physiological data in tandem with clinical information

Our study explored the PPCs’ predictive performance of three different data inputs: physiologic parameters, clinical parameters, and physiologic parameters combined with clinical parameters, respectively. Multiple classifiers showed predictive power when clinical features were used as inputs alone, with AUCs above 0.7 and acceptable performance for ACC, F1, and precision. This observation confirms the importance of clinical data, consistent with the findings of numerous researchers [3]. The classification performance was not good, with an AUC of around 0.6, when physiological features alone were used as inputs. However, the predictive performance was improved by an average of 8.32%, 1.80%, 3.28%, 6.06% in AUC, ACC, F1, and precision when physiological features were combined with clinical features as inputs across classifiers. Moreover, DeLong test results showed that the integration of physiological and clinical data significantly enhances the performance of machine learning models, including XGB, Random Forest, and K-Nearest Neighbors, in the preoperative risk assessment of PPCs compared to using single datasets. These demonstrated the ability of physiological data to add additional information to the model for preoperative identification of PPCs, and also highlighted the great potential of such highly individualized, long-term, continuous data captured by wearable devices for longitudinal data analysis and predictive analyses of disease [28]. Our research suggested that combining wearable and clinical data provides a more comprehensive approach to predicting individual states.

### A machine learning model for preoperative PPCs risk assessment

Our results showed that XGB outperforms other machine learning algorithms in constructing risk prediction models for PPCs. A recent study emphasized the effectiveness of XGB within ensemble models for cardiac surgery risk prediction, demonstrating improved predictive accuracy and variable interpretability [29]. This further supports our conclusion that XGB is a robust tool for handling complex datasets and capturing non-linear relationships, making it valuable for developing high-performance health risk prediction models. Additionally, the interpretability of XGB is crucial for understanding variable importance and enhancing clinical application.

Our study identified eight PPCs risk factors, including four clinical factors and four physiological characteristics. Univariate logistic regression showed that each of the eight features, when used individually, yielded an AUC greater than 0.6 for PPCs. Additionally, surgical methods, age, nn_50, and min_ven_in_mean were found to be significantly associated with the occurrence of PPCs.

Among all the features selected, the surgical method ranked first in importance. As SAVR requires a sternotomy, patients face large surgical trauma, prolonged recovery, and lengthy hospitalization. Compared to traditional SAVR, TAVR adopts transcatheter heart valve replacement or repair surgery, which has the characteristics of not requiring extracorporeal circulation and cardiac arrest, less trauma, and quicker postoperative recovery. Postoperative pain also varies between surgical approaches, with the site of open-heart surgery dictating that postoperative pain will inhibit patient breathing and forceful coughing to some extent [30]. The significant differences between the two surgical methods may lead to different PPCs risks, consistent with the results of numerous studies [5, 6, 9]. Also, its ranking importance suggests that we should develop separate PPCs risk prediction models for the different surgical approaches in follow-up work.

Elderly age is one of the high-risk factors for many surgical procedures [14] and a cause of respiratory failure [31]. They typically face higher surgical risks and poorer outcomes because of frailty and reduced cardiorespiratory fitness. MIP is a frequently assessed data to detect inspiratory muscle strength. High preoperative prevalence of inspiratory muscle weakness in patients undergoing elective cardiac surgery has been reported to be significantly associated with a high risk of PPCs [32] and prolonged mechanical ventilation [33]. Preoperative inspiratory muscle training (IMT) significantly improves postoperative MIP, promotes recovery of lung function, and reduces the risk of pulmonary complications after cardiothoracic or upper abdominal surgery by approximately 50% [34]. The dPA reflects the degree of pulmonary artery dilatation. Increased dPA is a feature of pulmonary hypertension, and preoperative pulmonary hypertension is a known predictor of increased mortality in TAVR patients with severe aortic stenosis. The dPA > 29.3 mm was associated with higher 1-year mortality after TAVR [35].

Rem_per is commonly used in polysomnography (PSG) to assess sleep disorders in the surgical population and reflects the quality of a patient’s sleep. Min_ven_in_mean provides information on the dynamics of breathing during sleep. OSA is considered one of the risk factors for PPCs [15, 36]. REM sleep-related OSA, one of the most common sleep-related breathing disorders, worsens cardiac autonomic function and is very common in patients undergoing cardiovascular surgery [37]. RSBI_in_mean has been commonly used to predict weaning failure with a cut-off of 105 breaths/min/L [38]. Patients with a lower RSBI are more likely to be weaned successfully [39]. Inversely, a high RSBI leads to progressive hypoventilation by increasing dead space ventilation [40], which further suggests impaired lung function. The nni _ 50 indicates cardiac rhythm. About 90% of tricuspid valve replacement patients may have atrial fibrillation before surgery [41], which may lead to a worse prognosis [42].

Combining multi-parameter wearable monitoring devices, clinical medical records and machine learning algorithms may present an opportunity to reveal the risk of PPCs in the valve surgery population at an early preoperative stage. Compared to traditional preoperative screening methods for PPCs that rely on patient history, clinical questionnaires or scales, and static clinical examinations, our approach involves a more detailed dynamic assessment by incorporating low-load, comprehensive physiological indicators. The Risk factors included in the model are common, easily accessible, and have good interpretability, providing new ideas for PPCs risk prediction. Identifying high-risk patients preoperatively facilitates optimized surgical management, prevents PPCs, and reduces patient morbidity and mortality. The portability of the wearable device and its potential to enhance surgical care represent a significant advancement, promising to improve patient outcomes and support clinical decision-making through real-time monitoring.

### Limitations

There are still some limitations in this study. First, this research is a single-center exploratory research with a moderate sample size. In the past years, due to the medical resource relocation for the unforeseen epidemic control, only 100 cases of 24-hour continuous physiological data were obtained in this study. Another challenge is that patients were admitted for various clinical examinations and often interrupted, making this sample size moderate but invaluable. On this sample set, we conducted an exploratory study of nocturnal sleep stages, confirming that predicting PPCs risk preoperatively using wearable devices is feasible. Further work will investigate the clinical value of 24-hour continuous physiological data, increase the sample size, and conduct a prospective multi-center observational study involving different regions and population characteristics in China.

Secondly, this study found that surgical methods have a crucial impact on the risk of PPCs. To adequately reflect advances in surgical technology, there is a need to establish respective predictive models for different surgical methods as the number of valve replacement surgeries increases. In addition, it is necessary that the model undergoes more external validation before it can be widely applied in clinical practice, especially among hospitals whose surgical populations have mild or moderate severity of heart valve disease and lower risks of postoperative complications. Hence, further work will focus on refining the model by fine-tuning the limited dataset from a specific hospital, taking into account factors such as disease severity and PPCs incidence, and improving the accuracy of the model through transfer learning or other methods. Moreover, presented in risk calculation software or an online calculator, the model would be more easily applied in clinical practice.

## Conclusions

Our study confirms the feasibility of using continuous physiological data collected by wearable devices to predict a patient’s risk of developing PPCs after valve surgery. Combining of continuous physiological data, clinical data and the XGBoost algorithm yields a well-performing and interpretable model. The results are expected to provide clinicians with an easy-to-use tool to identify people at high risk of PPCs before valve surgery, hoping to prompt targeted prehabilitation.

## Electronic supplementary material

Below is the link to the electronic supplementary material.

Supplementary Material 1

## Data Availability

The datasets presented in this article are not readily available because patient privacy needs to be protected. Requests to access the datasets should be directed to contact the Department of Cardiovascular Surgery, West China Hospital, Sichuan University. The source code for data preprocessing and model development has been made publicly accessible on GitHub and can be viewed at this URL: https://github.com/Preoperative-assessment-of-PPCs-risk/PPC.
